# The central role of aquaporins in the pathophysiology of ischemic stroke

**DOI:** 10.3389/fncel.2015.00108

**Published:** 2015-04-08

**Authors:** Jasmine Vella, Christian Zammit, Giuseppe Di Giovanni, Richard Muscat, Mario Valentino

**Affiliations:** Department of Physiology and Biochemistry, University of MaltaMsida, Malta

**Keywords:** aquaporin (AQP), ischemia, edema, glutamate, astrocytes, glial scar, calcium signaling, K_ir_4.1

## Abstract

Stroke is a complex and devastating neurological condition with limited treatment options. Brain edema is a serious complication of stroke. Early edema formation can significantly contribute to infarct formation and thus represents a promising target. Aquaporin (AQP) water channels contribute to water homeostasis by regulating water transport and are implicated in several disease pathways. At least 7 AQP subtypes have been identified in the rodent brain and the use of transgenic mice has greatly aided our understanding of their functions. AQP4, the most abundant channel in the brain, is up-regulated around the peri-infarct border in transient cerebral ischemia and AQP4 knockout mice demonstrate significantly reduced cerebral edema and improved neurological outcome. In models of vasogenic edema, brain swelling is more pronounced in AQP4-null mice than wild-type providing strong evidence of the dual role of AQP4 in the formation and resolution of both vasogenic and cytotoxic edema. AQP4 is co-localized with inwardly rectifying K^+^-channels (K_ir_4.1) and glial K^+^ uptake is attenuated in AQP4 knockout mice compared to wild-type, indicating some form of functional interaction. AQP4-null mice also exhibit a reduction in calcium signaling, suggesting that this channel may also be involved in triggering pathological downstream signaling events. Associations with the gap junction protein Cx43 possibly recapitulate its role in edema dissipation within the astroglial syncytium. Other roles ascribed to AQP4 include facilitation of astrocyte migration, glial scar formation, modulation of inflammation and signaling functions. Treatment of ischemic cerebral edema is based on the various mechanisms in which fluid content in different brain compartments can be modified. The identification of modulators and inhibitors of AQP4 offer new therapeutic avenues in the hope of reducing the extent of morbidity and mortality in stroke.

## Introduction

Stroke is one of the primary causes of death in many developed countries, with a slow increase in prevalence worldwide (Kim et al., 2010). The most common type, ischemic stroke, accounts for approximately 85% of deaths and occurs when reduced cerebral blood flow caused by acute or gradual vessel obstruction results in damage to a local area of the brain (Green et al., 2003; Hossmann, 2006). The pathophysiology of ischemic stroke integrates several complex molecular and hemodynamic processes of the neurovascular unit and a sound understanding of the details of these events is required if treatment design strategies are to be successful.

Aquaporins (AQPs) are a family of specialized water channels expressed on heterogeneous cell types, of which at least three major types are found in the central nervous system (Papadopoulos et al., 2007). Their discovery has greatly improved our knowledge on how water flux is absorbed and released across various membranes, and has provided insight on water distribution in the various brain compartments (Agre, 2004). They contribute significantly to water homeostasis in the body and have important roles in both physiology and in heterogenous diseases (Papadopoulos et al., 2007).

Since normal brain function is intricately linked to water homeostasis (Amiry-Moghaddam and Ottersen, 2003c), it is not surprising that AQPs have taken center stage in studies related to various brain diseases since their discovery. With pathophysiological features including edema and inflammation, ischemic stroke is one of such diseases deserving attention. Given that edema is one of the principal mediators of ischemic injury, this review aims to highlight recent advances in knowledge of AQP channels in the brain, and on their putative roles in the evolving pathophysiology of ischemic stroke. The development of pharmacological tools targeting AQPs to reduce edema and mitigate glial scar formation will also be addressed.

## Ischemic stroke and cerebral edema

**Figure 1:**
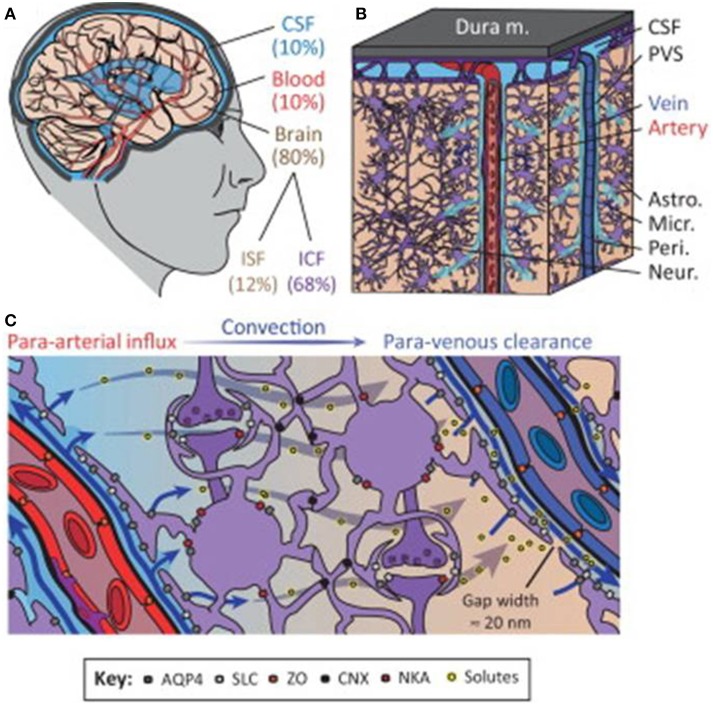
**The glymphatic system regulates cerebrospinal fluid (CSF) and interstitial fluid (ISF) exchange in the brain. (A)** Illustration of the main fluid compartments in the brain. **(B)** Diagram of fluid influx via penetrating arteries and efflux along a subset of large-caliber veins. **(C)** Diagram of proposed molecular mechanisms governing paravascular CSF–ISF exchange. Abbreviations: paravascular space, PVS; solute carrier, SLC; zonula occludens, ZO; connexin, CNX; Na^+^-K^+^-ATPase, NKA; intracellular fluid, ICF; aquaporin-4, AQP4. Reproduced with permission from Thrane et al. (2014).

In the adult brain, water is distributed among various compartments that include the cerebrospinal fluid (CSF), blood, and the intracellular and interstitial components of the parenchyma. Fluid movement across vascular, ventricular and parenchymal compartments is controlled by osmotic gradients and hydrostatic pressure differences and is crucial for maintaining normal physiological function (Papadopoulos et al., 2007). The neurovascular compartment consisting of vasculature, neurons and astrocytes is critical for the control of this water movement (Badaut et al., 2011). The recently coined “glymphatic system” by Iliff and Nedergaard (reviewed in Iliff and Nedergaard, 2013), attempts to further delineate water movement facilitated via a paravascular route between glial and vascular cells that may include previously unrecognized pathways (Figure 1). In ischemic stroke, a key aggravating factor is the presence of edema. Brain volume is limited by the rigidity of the skull, so that even minute increases in volume lead to an increase in intracranial pressure and compression of neural tissue and vasculature. Edema therefore has a crucial impact on morbidity and mortality in stroke (Klatzo, 1985). Current treatment of cerebral edema is limited to osmotic agents such as mannitol and surgical decompression, but these do not address the problem at a molecular level.

## Edema formation in stroke pathophysiology

In ischemic stroke, edema is commonly observed within the first week, reaching a peak 24–72 h following the ischemic event (Marmarou, 2007) and is usually accompanied by alterations in the neurovascular unit at the molecular and cellular levels (Badaut et al., 2011). Ischemic brain edema can be differentiated pathophysiologically into an early cytotoxic form, arising a few minutes following ischemia and a later vasogenic type appearing as a result of blood-brain barrier (BBB) breakdown after around 4–6 h. More recent denominations including anoxic and ionic edema focus on the etiology of the edema rather than on the location of its effects (Simard et al., 2007; Badaut et al., 2011).

Ischemic edema follows a characteristic time course and is vascular threshold-dependent. It first manifests at perfusion values of ~30% of control when stimulation of anoxic metabolism causes an increase of brain tissue osmolarity and associated cell swelling. At flow values below 20% of control anoxic depolarization and failure of cellular ionic regulation further elevate intracellular osmolarity and the associated cell swelling (Hossmann, 2006). During this acute phase, tissue injury is the direct consequence of ischemia-induced energy failure and depolarization consequent to the cessation of Na^+^/K^+^ -ATPase and Na^+^ co-transporter. Intracellular accumulation of ions and failure of reuptake mechanisms results in disruption of ionic gradients (Choi and Rothman, 1990). Osmotic water influx causes astrocyte and dendritic swelling (Kimelberg, 2005), and a diminution of the extracellular compartment, but does not alter the net water content (Nicholson and Sykova, 1998). The shift of fluid is reflected by a decrease in the apparent diffusion coefficient of water, which is the reason for the increase in signal intensity in diffusion-weighted MR imaging (Hoehn-Berlage et al., 1995). Physiologically, astrocytes are capable of rapidly responding to changes in extracellular osmotic pressure by an initial swelling and consequent regulatory volume decrease. This regulatory volume decrease is controlled by ion and water movements and is thought to be under the control of volume-activated K^+^-and Cl^−^-channels (O'Connor and Kimelberg, 1993). In ischemia, these mechanisms may still function but may be overwhelmed, which hampers the ability of astrocytes to maintain their volume and water content. Cellular edema is primarily a pathology of astroglia, and astroglial swelling becomes apparent within 30 min of onset of focal ischemia (Pantoni et al., 1996). The initiation of astrocyte swelling is probably largely associated with increased levels of glutamate and K^+^ which activate astrocyte-mediated uptake and trigger cellular osmotic overload. Subsequent activation of water influx through AQPs, which are expressed abundantly in astrocyte membranes, results in a dramatic increase in their volume (Nielsen et al., 1997) which may also be augmented during hypoxia by lactacidosis (Walz and Mukerji, 1988).

Astroglial swelling can initiate a variety of secondary effects, which exacerbate brain damage. In particular, swelling of perivascular astrocytes and astrocyte end-feet may compress brain vessels and limit vascular circulation. Vascular congestion from this mechanism contributes to the incomplete filling of brain vessels during reperfusion following focal ischemia, known as the *no-reflow* phenomenon (Ames et al., 1968). Swelling of astrocytes can result in opening of volume-regulated ion channels which are permeable to glutamate and other excitatory amino acids whereas release of the latter can induce or exacerbate excitotoxic cell death. Prominent swelling of astrocytes can also severely reduce the extracellular space volume which contributes to a concentration of extracellular glutamate and K^+^. A several-fold reduction in extracellular space is sufficient to increase the concentration of extracellular glutamate to excitotoxic levels (Choi and Rothman, 1990).

With the development of tissue necrosis and the degradation of the basal lamina BBB integrity is lost and after 4–6 h albumin and other serum proteins begin to leak from the blood into the brain following disturbance of endothelial tight junctions (Wang and Shuaib, 2007). This event initiates a delayed vasogenic type of edema which enhances the water content of the tissue by more than 100%. In large brain infarcts, the volume increase of the edematous brain tissue may be so pronounced that transtentorial herniation causes compression of the midbrain. Under clinical conditions, this malignant form of brain infarction is by far the most dangerous complication of stroke and an indication for decompressive craniectomy (Walz et al., 2002). In a study of transient middle cerebral artery occlusion (MCAO) in cats, Toyota et al. (2002) showed that glutamate elevation during ischemia is not only a reliable predictor of secondary deterioration but also an important cause leading to a malignant course with decreased cerebral perfusion pressure. Toyota et al. (2002) hypothesized that glutamate elevation may lead to infarct enlargement and further enhancement of glutamate efflux through positively controlled feedback mechanisms. The formation of cytotoxic and to a lesser extent of vasogenic edema requires flow of water through AQP channels located in the plasma membrane (Badaut et al., 2002). Inhibition of AQP water conductance at various stages during stroke may therefore reduce the severity of ischemic brain edema.

## Countering edema

Under physiological conditions, edema is efficiently cleared through translocation via the ependyma into the ventricular CSF, the glia limitans into the subarachnoid CSF, and through the BBB into the blood. The elements of this exit route strongly express the AQP4 transporter and the relative contribution of each to resolution of edema may depend on the surface area of each barrier and the intracranial pressure (Tait et al., 2008).

**Figure 2:**
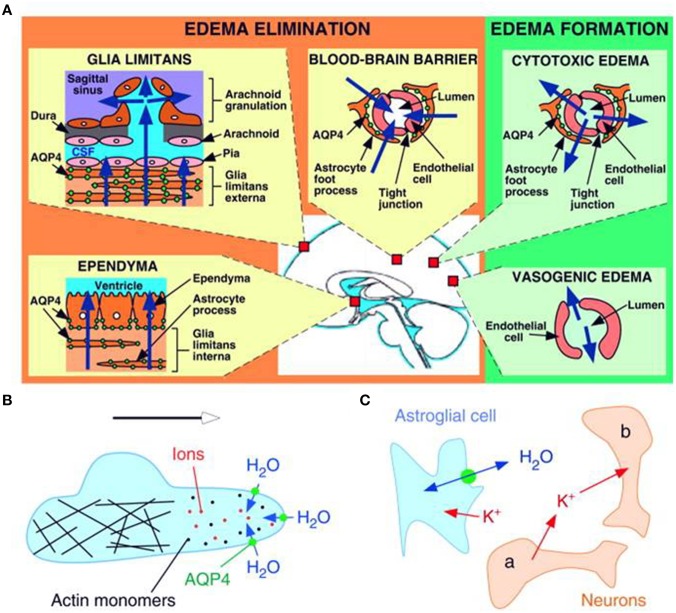
**Schematic drawing depicting three distinct roles of AQP4 (green circles) in brain function. (A)** Brain water balance, **(B)** astroglial cell migration and **(C)** neuronal excitation. **(A)** Green. Routes of edema formation in the two types of brain edema (cytotoxic – through AQP4, vasogenic – through interendothelial spaces). Orange. Edema fluid is eliminated by AQP4 through the glial limitans into subarachnoid CSF, through ependyma and sub-ependymal astroglia into ventricular CSF, and through astroglial pericapillary foot processes into blood. **(B)** AQP4 polarizes to the leading edge of migrating astroglia and accelerates cell migration. AQP4 facilitates water entry into lamellipodial protrusions in response to intracellular hyperosmolality produced by actin depolymerization and ion influx. **(C)** AQP4 deletion reduces neuroexcitation. Active neurons (neuron a) release K^+^ into the extracellular space (ECS). Increased extracellular [K^+^] depolarizes quiescent neurons (neuron b). AQP4 deletion increases ECS volume and reduces astroglial cell K^+^ reuptake. This buffers the increase in extracellular [K^+^] by active neuron a, preventing depolarization of quiescent neuron b. Reproduced with permission from Papadopoulos and Verkman (2008).

Vasogenic edema has traditionally been thought to be cleared primarily by bulk flow of fluid through the extracellular space, through the glia limitans into the ventricles and subarachnoid space, and to a lesser extent through astrocyte foot processes and capillary endothelium into the blood. Extravasation of albumin protein following BBB breakdown further increases the bulk flow of water and edema in the extracellular compartment of the brain. In a series of experiments conducted in AQP4-null mice, Papadopoulos and colleagues have shown strong evidence that AQP4-dependent transcellular water flux is central to the movement of edema fluid across the astrocyte cell membranes of the glia limitans into the CSF (Papadopoulos et al., 2004). These findings support earlier results by Reulen and colleagues, who demonstrated movement of edema fluid toward the ventricle (Reulen et al., 1978). There have been several reports of altered AQP4 expression in astrocytes in cases of brain edema in both human and rodent brain (reviewed in Papadopoulos and Verkman, 2013 and others). The severity of the lesion producing interstitial edema was associated with the up-regulation of AQP4 and this could potentially be a protective mechanism for countering edema accumulation (Tourdias et al., 2009). Thus, the up-regulation of AQP4 expression could be an important determinant of the overall water content on the basis of its involvement in the elimination of edema from the brain parenchyma (Figure 2A).

## The aquaporin channel family

**Figure 3:**
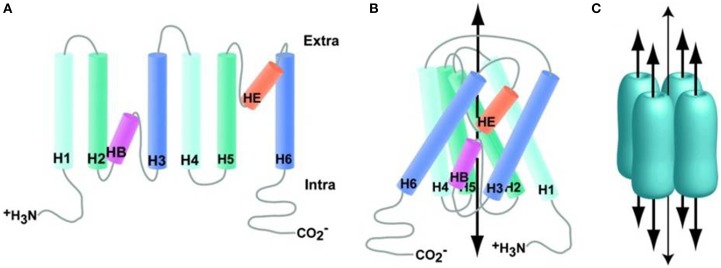
**Aquaporin structure. (A)** Generalized schematic of AQP family proteins expanded to show connectivity. **(B)** Generalized schematic of AQP family protein structure collapsed to show protein folding, wide arrow indicates the approximate substrate path. **(C)** Generalized schematic of AQP biological unit, wide arrows indicate substrate path; the narrow arrow indicates the proposed path of dissolved gasses through the central pore. Reproduced with permission from Huber et al. (2012).

The members of the AQP water channels consist of small (~30 kDa/monomer) hydrophobic, integral membrane proteins. These are expressed widely in the animal and plant kingdoms with 13 subtypes having been identified so far in mammals. The existence of a channel pore for water was hypothesized before the identification of the protein and was based on observations that red blood cells have higher water permeability than expected for an equivalent surface area of a lipid bilayer membrane. This hypothesis was confirmed in 1992 by Peter Agre and colleagues with the identification of AQP0, the first member of the AQP family of water channels (Preston et al., 1992). Besides functioning as a water channel, APQ0 also has a structural role, being required to maintain the transparency and optical accommodation of the ocular lens. The monomeric units of AQPs consist of six transmembrane α-helices (M1, M2, M4–M7, and M8), two half helices (M3 and M7) and five connecting loops around a water pore (Figure 3). Their specificity for water may depend on two conserved Asn-Pro-Ala (NPA) motifs in the half helices M3 and M7, which contain inward-facing asparagine polar side chains that prevent proton conduction (Murata et al., 2000). Together with the α-carbonyl groups, the NPA motifs act as hydrogen-bond donors and acceptors that allow the transport of water or glycerol through the pore (Fu et al., 2000; Sui et al., 2001). Molecular simulations based on the AQP1 crystal structure suggest single-file passage of water through the narrow <0.3 nm pore, in which steric and electrostatic factors prevent transport of protons and other small molecules (Sui et al., 2001). AQPs have no gating system for water permeability through their channel pore and therefore functions of APQs depend on their level of expression in the plasma membrane. The transport function of many AQPs can be inhibited by non-specific, mercurial sulfhydryl-reactive compounds, such as mercury chloride and there is growing interest in the design of non-toxic, AQP-selective inhibitors (Verkman, 2001; Castle, 2005).

These water-conducting, protein-based channels are divided into 2 major groups, on the basis of their permeability. AQP0, AQP1, AQP2, AQP4, AQP5, AQP6, and AQP8 comprise the pure water channel family and appear to be exclusive water channels. AQP6 and AQP8 are strictly in this group on the basis of sequence analysis, although AQP6 is permeated by anions (Yasui et al., 1999) and AQP8 might be permeated by urea and water (Ishibashi et al., 1997; Ma et al., 1997). Members of the second group include AQP3, AQP7, AQP9, and AQP10 known as aquaglyceroporins and are permeable to water, as well as glycerol and urea. AQP9 is permeable to water, glycerol, urea, purines, pyrimidines, and monocarboxylates (Badaut et al., 2002, 2011).

## Aquaporin distribution in the central nervous system

AQPs in the CNS facilitate water transport between the major brain compartments: the CSF space (including cerebral ventricles and subarachnoid space), the brain parenchyma (intracellular and extracellular space) and the intraventricular compartment (Zador et al., 2009; Papadopoulos and Verkman, 2013). In mouse brains, the AQPs that predominate in these compartments are mostly AQP1 and AQP4 (Nielsen et al., 1997). Only a subset of the 14 known AQPs, namely AQP1, AQP4 and AQP9, are expressed in the CNS. Each type of AQP has a unique expression pattern among tissues and during development factors such as injury may alter this expression. Sites of AQP expression may also give an indication to what function pertains to each type (Papadopoulos et al., 2007). Genetic defects involving aquaporin genes have been implicated in several human diseases. Most significantly, mutations in the AQP2 gene cause hereditary nephrogenic diabetes insipidus (Deen et al., 1994) and cataract formation with mutation in AQP0 (Verkman and Mitra, 2001).

## Aquaporin 4—the major aquaporin in the brain

**Figure 4:**
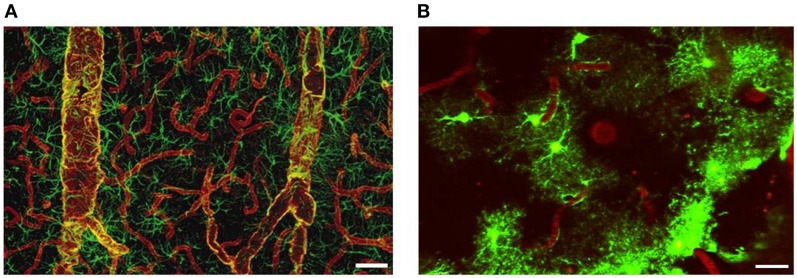
**Distribution of AQP4 and coverage of cortical astrocyte microdomains at the gliovascular interface. (A)** Double immunolabeling of AQP4 (red) and GFAP (green). AQP4 immunolabeling reveals that the entire network of vessels, including capillaries, is covered by astrocyte processes, albeit GFAP negative. Smaller vessels and capillaries are mostly GFAP negative but display intense labeling against the astrocyte-specific channel AQP4. The AQP4 labeling reveals continuous coverage by astrocyte end feet. Scale bar: 60 μm. Reproduced with permission from Society of Neuroscience by Simard et al. (2003). **(B)** Two-photon imaging of enhanced green-fluorescent protein (eGFP)-expressing astrocytes on the cortical surface in live mouse brain, illustrating the territorial astrocyte domains and the dense array of processes associated with the vasculature. The vasculature was labeled with Texas Red-dextran dye that labels the plasma and outlines the pial vasculature. Scale bar: 20 μm.

The glial membrane channel AQP4, previously named as the mercury-insensitive water channel by Verkman's group is the most abundant water channel in the brain (Papadopoulos and Verkman, 2007). It is predominantly localized in astrocyte perivascular end-feet and glial limiting membranes, at the border between the brain parenchyma and subarachnoid CSF and beneath the ependyma bordering the brain parenchyma and ventricular CSF (Papadopoulos and Verkman, 2007; Zador et al., 2009). The tight organization of astrocytes around the vasculature provides anatomical evidence that they establish exclusive territories to form very specific microdomains in brain gray matter. Such an association allows receptors and channels essential for the function of astrocytes to be densely concentrated at their vasculature. Vascular end-feet intimately plastered along the walls of larger vessels typically co-express AQP4 and glial fibrillary acidic protein (GFAP). By contrast, astrocyte end-feet in contact with capillaries are AQP positive but only occasionally GFAP-positive (Figure 4). AQP4 is not expressed in other CNS cell types such as neurons, meningeal cells or oligodendrocytes (Papadopoulos et al., 2007), but has been reported in microglia following lipopolysaccharide (LPS) injection (Tomás-Camardiel et al., 2004).

Freeze-fracture and electron microscopy have shown the glia limitans to contain orthogonal arrays of particles (OAPs) or microcrystalline assemblies of AQP4 (Agre et al., 2002; Amiry-Moghaddam et al., 2010). The latter was confirmed through studies using transgenic mice which revealed that brains of AQP4-null mice were devoid of OAPs and from immunogold labeling of OAPs in the brain (Manley et al., 2000). These OAPs may play a central role in cerebral water balance and the association of AQP4 to OAPs has been suggested as augmenting its efficiency as a water transporter by increasing its water permeability and aiding its interaction with other proteins such as ion channels (Manley et al., 2000; Silberstein et al., 2004). AQP4 exists in two isoforms of which M1 occurs as individual tetramers and M23 forms OAP assemblies (Furman et al., 2003). The ratio between the expression of the long and the short AQP4 splice variant (AQP4m1 and AQP4m23) determines the size of the OAPs (Rash et al., 2004). The AQP4m23 isoform stabilizes the structure of the OAPs and an increase of its expression induces an increase in the size of the OAPs (Rash et al., 2004). Co-immunoprecipitation has demonstrated association of AQP4 with the dystrophin protein complex which is essential for anchoring of AQP4 to astrocyte end-foot processes (Neely et al., 2001). α-syntrophin, a PDZ-domain containing protein of the dystrophin associated protein complex is partly responsible for AQP4 anchoring to end-feet membranes and contributes to its polarized expression (Puwarawuttipanit et al., 2006). Targeted disruption of the gene encoding α-syntrophin results in inconsistencies including loss of perivascular AQP4 (Vajda et al., 2002).

AQP4 has been ascribed several roles within the CNS. These include control of water flux, neural signal transduction and promotion of astrocyte migration (Tait et al., 2008). Its polarized location in proximity to the vasculature also suggests a role in gaseous exchange including that of O_2_, CO_2_, and NO (Kimelberg and Nedergaard, 2010). Since it is expressed in cell types which perform an osmoregulatory role, AQP4 is thought to be a central modulator of serum osmolarity and although some of its roles remain controversial, strongest evidence points to AQP4 function in edema (Manley et al., 2000; Verkman, 2005).

Studies using AQP4- knockout mice (AQP-KO) and wild-type have aided in the elucidation of various roles of AQP4 in ischemia (Papadopoulos and Verkman, 2007). Considering the increased ratio of astrocytes to neurons from rodent to human brain, AQPs may play a more significant role in human than in rodent CNS (Papadopoulos and Verkman, 2013).

## AQP4 and edema formation

Astrocytes, being the dynamic, self-organizing, auto-regenerative housekeeping brain cells regulate several brain processes and the presence of heterogeneous transporters, receptors and ion channels are testament to their significance in cerebral homeostasis. Polarized AQP4 channel expression with high levels on perivascular astrocyte end-feet (Amiry-Moghaddam et al., 2003a), suggests a homeostatic role in water exchange between brain and vasculature. The role of AQP4 in ischemia was first described in an AQP4-KO model of combined cytotoxic and vasogenic brain edema (ischemic stroke model) and a cytotoxic brain edema model (acute water intoxication model) (Manley et al., 2000). In the ischemic stroke model, the presence of AQP4 was shown to aggravate post-ischemic cytotoxic edema as measured by post-ischemic hemispheric enlargement, while AQP4-KO mice exhibited an opposing effect and an improved neurological outcome (Manley et al., 2000; Papadopoulos et al., 2007). In the acute water intoxication model, AQP4-null mice had markedly reduced mortality from hyponatremia compared to wild-type mice. The protection afforded by the absence of AQP4 may be linked to reduced BBB water permeability and a reduced rate of water flux into the brain parenchyma (Papadopoulos and Verkman, 2007). In α-syntrophin-KO mice, deletion of AQP4 reduced hypo-osmolar edema following experimental stroke more than in wild-type mice (Amiry-Moghaddam et al., 2003a). Taken together, these results indicate that AQP4 mediates osmotically-driven water transport that follows ischemia (Manley et al., 2000; Badaut et al., 2011). In cytotoxic edema, this channel limits the rate of brain water accumulation by attenuating water flux from the vasculature into the brain parenchyma in the presence of an intact BBB (Manley et al., 2000; Papadopoulos and Verkman, 2013).

It is of interest that in vasogenic edema, AQP4 plays an opposing role. In established models of BBB disruption such as cortical freeze injury, brain tumor, brain abscess (Bloch et al., 2005), subarachnoid hemorrhage (Tait et al., 2010) and status epilepticus (Lee et al., 2012), AQP4-null mice develop more edema than do wild-type mice. This increased vasogenic component from deletion of AQP4 reaffirms that vasogenic elimination of water into the CSF and blood occurs via AQP4-dependent routes (Saadoun and Papadopoulos, 2010; Papadopoulos and Verkman, 2013).

AQP4 mediates bidirectional water flux, facilitating water influx in the evolution of cytotoxic edema and water efflux during edema elimination (Papadopoulos et al., 2007). Its increased expression at astrocyte end feet allows for polarization of function such that influx in one domain is associated with efflux in another (Nagelhus et al., 2004). This bimodal role is fundamental in formulation of therapeutic agents that target AQP4 in edema. For instance, AQP4 inhibition could provide new treatments by reducing cytotoxic edema during early ischemia, whereas vasogenic edema that appears in late ischemic stroke or hemorrhagic stroke could be potentially controlled by increased expression of AQP4, AQP4 up-regulators or activators (Papadopoulos and Verkman, 2007). Heterogeneous AQP4 expression patterns and their alterations in pathological states however add to the complexity of tackling the pathophysiology in ischemic stroke. Recent analyses revealed that integrins are lost and the astrocyte end-feet detach from the basal lamina soon after onset of ischemia (Tagaya et al., 2001; Kwon et al., 2009). This may affect astrocyte polarization and contribute to the pathophysiological events.

## AQP4 expression in ischemic stroke

AQP4 expression has been demonstrated to coincide with areas of glial-specific swelling (Manley et al., 2000), and their distribution is spatially and temporally regulated depending on the stroke model being employed (Badaut et al., 2011). In a mouse model of transient cerebral ischemia, AQP4 expression was rapidly up-regulated in perivascular end-feet, reaching a peak 1 h post-occlusion and coinciding with early cerebral swelling in the core and border of the lesion. Another peak of expression was observed in the penumbra after 48 h and was also correlated with the degree of brain swelling (de Castro Ribeiro et al., 2006). This finding may suggest that AQP4 could be the major water channel involved in water movements after transient cerebral ischemia. However, in models of more severe stroke, such as permanent distal MCAO, the early expression of AQP4 was not observed, suggesting that the brain is incapable of furnishing sufficient AQP4 proteins in more dire situations (Badaut et al., 2011). Differences in expression profile were also found in hypoxia-ischemia in young rats (Meng et al., 2005) and after MCAO of 90 min duration (Amiry-Moghaddam et al., 2003a). These landmark experiments showed that spatiotemporal up-regulation of AQP4 is multi-factorial and dependent on differences between stroke model and type, species and age, and severity of the insult.

Expression patterns of AQP4 vary significantly in different brain regions as a result of injury (Badaut et al., 2002). In a model of MCAO and reperfusion, perivascular AQP4 expression decreased significantly in the core area of the striatum after 24 h of ischemia-reperfusion. In contrast, AQP4 expression in the core of the cortex decreased after 24 h and recovered gradually after 72 h of reperfusion. This indicates that AQP4 may regulate fluid distribution between brain parenchyma and blood in the formation and dissipation of cerebral edema after ischemia-reperfusion (Li et al., 2008).

Early induction or down-regulation of AQP4 are also seen in various pre-conditioning experiments, in which application of sub-toxic or noxious stimuli aid in identification of potentially protective and regenerative endogenous mechanisms (Dirnagl et al., 2009; Hirt et al., 2009a; Hoshi et al., 2011). The involvement of astrocytes in synaptic function and plasticity via integration, processing and storage of information enables proper control of the brain homeostatic milieu. Since astrocytes are principal components of the brain's adaptive processes, the relationship of astrocyte AQP4 expression patterns to pre-conditioning stimuli seems to offer important mechanistic insights (Hoshi et al., 2011). In a thrombin pre-conditioning (TPC) model, Hirt and colleagues showed that exposure to the serine protease prior to ischemia was neuroprotective. Through immuno-histochemical and Western blot analysis, AQP4 expression was more pronounced in TPC mice than in saline-treated mice and this was correlated with reduced edema at 1 h post-stroke (Hirt et al., 2009b). Evidence of down-regulation of AQP4 was also seen with the use of 3-nitropropionic acid (3NP). This mitochondrial toxin at sub-toxic levels may induce ischemic tolerance through the inhibition of succinate dehydrogenase in the tricarboxylic acid cycle and may lead to the generation of reactive oxygen species (ROS). Administration of 3NP to rats prior to ischemia resulted in down-regulation of AQP4 and suppression of post-ischemic edema. The mechanism for this down-regulation may involve ROS-induced alteration of signal transduction through the glutathione antioxidant defense of astrocytes or by direct modification of the reversible oxidation of AQP4 (Hoshi et al., 2011). However, these findings need to be corroborated by further studies.

AQP4 expression in cultured astrocytes is enhanced by lactic acidosis induced by ischemia which activates the anaerobic glycolytic pathway for synthesis of ATP. Lactic acid causes swelling by accumulation of Na^+^, Cl^−^ and water accumulation via the coupled operation of Na^+^/H^+^ and Cl^−^/HCO^−^_3_antiporters, and the H^+^ monocarboxylate symporter. This increased AQP4 expression occurs at the membrane surface, without changes in mRNA level, suggesting redistribution or post-translational modification as key players of AQP over-expression (Morishima et al., 2008). Putative mechanisms of AQP4 modulation of cortical spreading depression (CSD) in stroke remain unclear.

## AQP4 and K_ir_4.1

In ischemic stroke, energy failure leads to ionic imbalance from modification of ionic gradient between intracellular and extracellular compartments. Primary active transport or secondary active transport leads to a net intracellular accumulation of Na^+^ followed by Cl^−^ and entry of osmotically-driven water, all of which contribute to the initial cytotoxic swelling in stroke (Simard et al., 2007). Increased extracellular K^+^ leads to the depolarization of neurons and glia, which in turn lead to increased efflux of glutamate. At this stage, glutamate-induced water influx and K^+^ clearance become additional causative factors of astrocyte end-foot swelling (Pa Santes-Morales and Cruz-Rangel, 2010).

K_ir_4.1 and K_ir_5.1 are inwardly rectifying K^+^ channels exhibiting distinct features (Pessia et al., 2001; Casamassima et al., 2003; D'Adamo et al., 2011). The location of glial AQP4 and their proximity to K_ir_4.1 has led to suggestions that AQP4 might aid in transport of K^+^ and hence regulate rapid changes in [K^+^] that occur during neural signaling (Zhang and Verkman, 2008). Recent studies have shown that K_ir_4.1 channels are also part of the orthogonal arrays owing to their expression in astrocytes (Kimelberg and Nedergaard, 2010) and further support the concept of K^+^ spatial buffering or siphoning as first documented in retinal muller cells (Newman et al., 1984). Disruption of the α-syntrophin gene leads to perturbed K^+^ homeostasis, further indicating the close association between K_ir_4.1 and AQP4, and the involvement of astrocyte end-feet in K^+^ homeostasis (Amiry-Moghaddam et al., 2003b).

During neuronal excitation, K^+^ is released into the ECS and efficient K^+^ clearance is required to prevent interference with neuronal signaling. The astrocyte AQP4 allow efficient uptake of water after intracellular K^+^ uptake and cause ECS shrinkage, produce hyperexcitability and enhance epileptiform activity (Papadopoulos and Verkman, 2013). This shrinkage may also occur if depolarization by K^+^ results in increased intracellular osmolarity through the Na^+^/HCO^−^_3_ cotransporter, which is strongly expressed on astrocytes (Nagelhus et al., 2004). In the absence of AQP4, alterations in [K^+^]_o_are buffered by small changes in ECS volume, since less water would be taken up into astrocytes. This expansion coupled to the reduced K^+^ siphoning alters neuronal excitability and lowers susceptibility to seizure (Tait et al., 2008). Deletion of AQP4 is also associated with impaired K^+^ reuptake as evidenced by altered seizure threshold and duration in AQP4-null mice compared to wild-type (Binder et al., 2006). Despite this association, confounding theories regarding the APQ4-K_ir_4.1 relationship abound (Figure 2C).

Direct *in-vivo* evidence of a decreased cellular re-uptake of K^+^ from the ECS in AQP4-null mice compared to wild-type has been reported (Verkman et al., 2006). However, later work presented significant evidence against functional interaction of glial AQP4 and K_ir_4.1 (Zhang and Verkman, 2008). In the latter study, there was no significant difference in membrane potential, K_ir_4.1K^+^ current or current-voltage curves of glial cells of AQP4 null or wild-type mice, nor any difference in K_ir_4.1 expression or distribution. Taken together, these results suggest that other glial cells or ion transporters, whose expression is modified by the absence of AQP4, are recruited to modulate local, K^+^ flux in neural signaling and excitation (Zhang and Verkman, 2008).

Another hypothesis implicates an association with K_ir_4.1, bicarbonate transporters and AQP4 flux. It has been suggested that during neuronal activity, active synapses lead to shrinkage of extracellular space accompanied by an increase in bicarbonate transport due to K^+^ accumulation (Nagelhus et al., 2004). This, coupled to secondary AQP4 water uptake may aid in the removal of CO_2_ and K^+^, thereby maintaining a balance between neuronal activity and metabolite clearance. Despite conflicting theories, APQ4 is still believed to play a key role in facilitating water uptake and redistribution of water from sites of neuronal activity to more distant sites, thus mediating activity-dependent volume changes and sustaining effective K^+^ clearance, whether directly or indirectly (Pellerin and Magistretti, 1994; Amiry-Moghaddam et al., 2003b; Nagelhus et al., 2004).

## AQP4 and altered calcium signaling in ischemic stroke

The original demonstration that astrocytes display a form of excitability based on changes in intracellular Ca^2+^ concentration (Cornell-Bell et al., 1990), prompted a re-examination of the actual role of astrocytes in brain function. Astrocytes show intracellular Ca^2+^ oscillations that occur spontaneously or in response to different neurotransmitters and that may serve as an intracellular and/or intercellular signal with relevant functional consequences in the nervous system (Pasti et al., 1997). Thus, Ca^2+^-mediated signaling is a critical mechanism by which astrocytes communicate and modulate the activity of adjacent cells (Pasti et al., 1997). Astrocyte Ca^2+^signaling is displayed in two modalities. Firstly, long-range propagating Ca^2+^ waves are evoked by electrical or mechanical stimulation, and by application of neurotransmitters such as glutamate or ATP. Secondly, Ca^2+^ oscillations describe an increase in cytosolic Ca^2+^ limited to a single cell. Exposure to various neurotransmitters including GABA, glutamate and ATP, removal of extracellular Ca^2+^ or exposure of cultured astrocytes to hypo-osmotic states are all conditions which give rise to these Ca^2+^ oscillations (Berridge et al., 1998).

The latter has been applied to acute cortical slices to allow for the observation of astrocyte-Ca^2+^ signaling events. Hypo-osmotic stress induces edema with early accumulation of astrocyte water (Nagelhus et al., 1993; Risher et al., 2009). This could offer a model for analysis of signaling cascades elicited by astrocytes *in vivo* and has been applied to assess the effect of AQP4 on astrocyte swelling and Ca^2+^ signaling (Thrane et al., 2011). In AQP4^+/+^ and AQP4^−/−^ mice exposed to mild hypo-osmotic stress (20% reduction in osmolarity), astrocyte swelling was observed in wild-type but was greatly reduced in AQP4^−/−^ mice. Ca^2+^ signals were only evoked in wild-type mice. More severe hypo-osmotic stress (30% reduction in osmolarity) produced astrocyte swelling and Ca^2+^ spikes in both wild-type and AQP4^−/−^ mice, suggesting an association between Ca^2+^ signaling, water influx via AQP4 and cellular swelling (Thrane et al., 2011). This result implies that Ca^2+^ signals may be evoked by AQP4-induced astrocyte swelling, rather than by AQP4 directly. Edema induces astrocyte swelling and this is augmented by the presence of AQP4. Swelling prompts release of toxic neuro-active substances such as glutamate and ATP which mediate aberrant Ca^2+^ signaling (Thrane et al., 2011). Thus, besides operating as an osmotic flux route, this AQP type may also trigger downstream signaling events and may exacerbate the pathological outcome. Modulation of AQP4 may aid in attenuating cell swelling and secondary effects such as deleterious ATP release. Activation of purinergic P2X7 receptors by ATP in repair and injury may also play an indirect role in modification of cerebral edema levels. In primary cultured astrocytes, P2X7 receptor activation significantly attenuated AQP4 protein expression. In addition, pre-treatment with P2X7 receptor antagonist in rats significantly inhibited AQP4 down-regulation in anoxic brain injury (Lee et al., 2008). This has led to the suggestion that purinergic receptor activation may confer a protective effect by attenuating cytotoxic edema through reduced water flux.

## AQP4 in regulatory volume decrease

Since AQP4 is predominantly expressed on astrocytes and water transport is inextricably coupled to major homeostatic processes served by these glial cells, control of the extracellular milieu by astrocytes may also involve this water channel type (Badaut et al., 2002; Nagelhus et al., 2004; Kong et al., 2008). In ischemic stroke, cytotoxic edema leads to neuronal and glial death with release of their contents into the extracellular space (Papadopoulos and Verkman, 2007). Swollen cells including astrocytes, undergo effective cell volume regulation by the phenomenon of regulatory volume decrease (Badaut et al., 2002; Kimelberg, 2005; Papadopoulos and Verkman, 2007). Extrusion of intracellular ions, such as K^+^ and Cl^−^ and amino acids including taurine and glutamate into the ECS with decrease of the cell size to baseline in the process has been described (Badaut et al., 2002; Papadopoulos and Verkman, 2007). These osmotically-active solutes are released with water so that AQP4 could be involved in the control of regulatory volume decrease (Badaut et al., 2002; Kimelberg, 2005). Down-regulation of AQP4 in rat cortical astrocytes attenuates the volume-sensitive Cl^−^ current evoked by hypo-osmolarity, indicating that these currents or the volume-regulated anion channels (VRAC) that mediate them play crucial roles together with water channels in regulatory processes that decrease volume (Pa Santes-Morales and Cruz-Rangel, 2010). Such volume decrease is critical in counteracting the detrimental swelling caused by pathophysiological insults in ischemic stroke. Information regarding functional interaction of these proteins, however, is still lacking while recent studies have reported that glutamate and other co-transporters expressed on astrocytes may facilitate water transport across areas where APQ4 is not expressed (MacAulay et al., 2004; Kimelberg, 2005).

## AQP4 and Cx43

Cx43, a primary gap junction protein (connexin) abundantly expressed in astrocyte end-feet, mediates cell-cell interaction by facilitating ionic and molecular transport (Rash et al., 2004) and has recently been implicated in brain damage-related abnormal neuro-differentiation (Samarasinghe et al., 2014). This allows for efficient activation of many neighboring astrocytes in a relatively short time and, with purinergic channels, has been implicated in the generation and propagation of astrocyte Ca^2+^waves (Cotrina et al., 1998; Arcuino et al., 2002). In AQP4-KO cultured mouse astrocytes, Cx43 expression was down-regulated (Nicchia et al., 2005), indicating an interaction between this connexin and water channel type.

Cx43 and APQ4 may work in tandem to allow water flux and movement of ions and small molecules from the brain parenchyma toward the vasculature. At perivascular end-feet these ions and small molecules become extruded via their respective channels. A prime example would be K^+^ siphoning when extracellular K^+^ and water may permeate through Cx43 and are extruded through Cx43 and AQP4 respectively (Nicchia et al., 2005). Simultaneous action of Cx43 and AQP4, together with K^+^ channels (K_ir_4.1/K_ir_5.1) would also ensure efficient removal of K^+^ from astrocytes into the vasculature (Lichter-Konecki et al., 2008). Indeed, increased extracellular levels of K^+^, as seen during ischemia, have been shown to increase gap junctional coupling and AQP4 expression (De Pina-Benabou et al., 2001).

Although the mechanisms are not clear, a functional relationship could exist, possibly by coordination of ion and water movements through Cx43 when AQP4-mediated flux is compromised (Nicchia et al., 2005; Chanson et al., 2007). Such a functional relationship could play a pivotal role in distribution of edema through astrocyte syncytial networks (Fukuda et al., 2013a).

## Effect of glutamate on AQP4

Under physiological conditions, glial glutamate uptake is achieved primarily through the major glutamate astrocyte buffering gateway in the brain–high-affinity Na-dependent transporters such as GLT-1 (EEAT2) or GLAST (EEAT1) (Nicholls and Attwell, 1990). Glutamate uptake via these transporters is accompanied by Na^+^ uptake and water influx aided by AQP4. Glutamate also activates group I metabotropic receptors (mGluR), which in turn activate phospholipase C (PLC) and generates inositol 1,4,5- triphosphate (IP_3_) triggering Ca^2+^ release from intracellular stores and activates calcium/calmodulin-dependent protein kinase II (CaMKII) and nitric oxide synthase (NOS). Group I mGluR also regulate water permeability of AQP4-expressing astrocytes (Gunnarson et al., 2008).

A link between glutamate release and AQP-4 expression has also been observed in acute rat hippocampal slices (Zeng et al., 2012). AQP4 knockout mice were found to decrease glutamate uptake by astrocytes via down-regulation of GLT-1, with increase in extracellular glutamate levels (Zeng et al., 2007, 2012). Excessive glutamate levels lead to activation of mGluR with increase in hypo-osmotic swelling in tissue slices (Zeng et al., 2012). AQP4 and GLT-1 in fact, localize as a functional complex on astrocyte membranes (Zeng et al., 2007). AQP4 expression correlated with the GLT-1 expression pattern while astrocytes positive for AQP4 were also shown to be positive for GLT-1 (Mogoanta et al., 2014).

In ischemia, an energy crisis leads to ionic disturbances with intracellular accumulation of Na^+^ and extracellular K^+^ and increased water influx from the extracellular space. As ionic gradients cannot be regulated efficiently in these conditions, astrocyte AQP4 may have a detrimental effect by facilitating water entry which aggravates cytotoxic edema. Excessive glutamate levels also up-regulate AQP4 astrocyte activity via mGluR activation, which further exacerbates the edematous insult (Gunnarson et al., 2008).

Ischemic brain damage ultimately leads to neuronal death by excitotoxicity so that the control of extracellular glutamate levels and prevention of excitotoxicity determine the degree of cell survival around the ischemic area (Choi and Rothman, 1990). AQP4 modulation by metabotropic receptors or signaling chain stimuli, particularly inhibition of AQP4 up-regulation, offers a possible therapeutic avenue to minimize brain edema from the excessive glutamate that is released during ischemia (Gunnarson et al., 2008; Pa Santes-Morales and Cruz-Rangel, 2010).

## AQP4, cell migration, and glial scar formation

The ability of astrocytes to form scars that inhibit axon regeneration and neuronal repair has been recognized for over a century. The astroglial defensive response is manifested by a series of co-ordinated processes that involve changes in the expression of genes and result in the appearance of multiple phenotypes specific to a particular pathology (Burda and Sofroniew, 2014; Pekny et al., 2014). Nevertheless, a growing body of evidence indicates that reactive astrocytes exert many beneficial functions and that astrocytes have a wide spectrum of potential and often subtle responses to CNS insults, scar formation being one that lies at the extreme end in terms of its severity. In particular, models of spinal cord injury have shown that the glial scar and reactive astrocytes may confer protective advantages to recovering CNS tissue which then progress into a maladaptive response that renders the tissue non-responsive and detrimental to repair. Studies of gene expression in astrocytes following MCAO in mice showed up-regulation of many of the proteins present in reactive astrocytes and glial scar following spinal cord injury. This suggests that the function of the glial scar in stroke would be similar to that in spinal cord injury (Zamanian et al., 2012). Inhibition of reactive astrogliosis generally reduces neuronal viability, affects nervous tissue regeneration, suppresses neurogenesis and compounds the neurological outlook (Robel et al., 2011; Burda and Sofroniew, 2014).

After an ischemic insult, reactive astrocytes become metabolically activated by augmenting their nuclear size and increasing the number of mitochondria and ribosomes. They migrate toward the lesion and remodel it to form a packed glial scar (Panickar and Norenberg, 2005). Accumulated glial filaments containing GFAP, vimentin and/or nestin are hallmarks of hypertrophic astrocytes during ischemia (Pekny and Nilsson, 2005; Sofroniew and Vinters, 2010). The appearance of reactive astrocytes with GFAP expression is concurrent with AQP4 up-regulation (Eng et al., 2000). The glial scar also contains reactive microglia which express AQP4 (Tomás-Camardiel et al., 2004).

Migration of reactive astrocytes is seen as an important component of glial scar formation around the damaged tissue and results of *in vitro* and *in vivo* experiments support the notion of AQP4-facilitated migration of reactive astrocytes toward the site of injury. An *in vivo* model of reactive gliosis and astroglial cell migration produced by cortical stab injury showed that glial scar formation was remarkably impaired in AQP4-null mice because of reduced migration of reactive astroglia toward the site of injury (Saadoun et al., 2005). Based on these findings, it has been proposed that AQP4 accelerates astroglial cell migration by increasing plasma membrane permeability to water, which increases the transmembrane water fluxes that take place during cell movement (Figure 2B). Osmotic gradients created by actin cleavage and ion uptake lead to water flux mediated by AQP4 which facilitates lamellipodial extension and speeds up cell migration (Verkman, 2005). High AQP4 expression may contribute to cell adhesion and facilitate adhesion between astrocytes so that a new barrier or the glia limitans is formed (Hiroaki et al., 2006; Badaut et al., 2011). Despite data still being fragmentary, these studies provide new insights into why APQ4 is up-regulated in astrocytes during ischemia, with no edema-related effects. Strategies aimed at AQP4 may help attenuate glial scarring and enhance neuronal regeneration (Saadoun et al., 2005).

## AQP4 and neuroinflammation

Ischemic stroke-related brain injury triggers inflammatory cascades that further amplify tissue damage. Neuroinflammation is usually accompanied by edema and BBB disruption. It is distinct from peripheral inflammation (Galea et al., 2007; Graeber et al., 2011), being characterized by microglial activation, reactive astrocytosis and activation of chemokines and cytokines. In cerebral ischemia this may be beneficial or detrimental, depending on the timing and severity of the insult.

**Figure 5:**
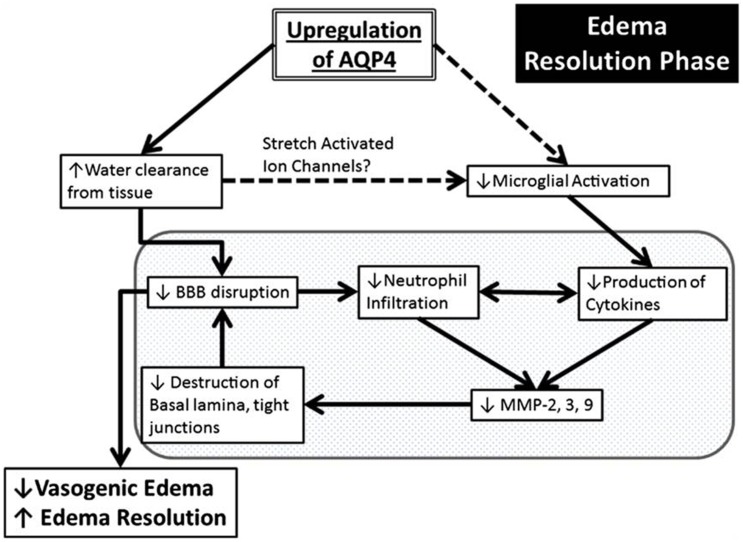
**Schematic summary of a beneficial role AQP4 upregulation plays during the edema resolution phase**. The upregulation of AQP4 causes increased water clearance from the tissue, which in turn causes decreased BBB disruption because of decreased pressure, and there is less neutrophil infiltration and decreased pro inflammatory cytokines. This causes decreased MMP production (Candelario-Jalil et al., 2009) which possibly results in less destruction of the basal lamina and tight junctions and causes an even greater decrease of the BBB. In another pathway (dotted lines), the increased water clearance from the tissue and extracellular space causes changes in the osmotic pressure, changing the activation state of the stretch activated ion channels expressed in microglia (Lewis et al., 1993; Eder et al., 1998; Schlichter et al., 2011), causing less microglial activation, leading to decreased pro-inflammatory cytokine release. The resulting decrease in BBB disruption/permeability leads to decreased vasogenic edema or better edema resolution. Finally, this scheme outlines the potential link between AQP4, edema and neuroinflammation. Reproduced with permission from Fukuda and Badaut (2012).

A central role of AQP4 in neuroinflammation is in the established astrocyte proliferative response in ischemia (Kuppers et al., 2008). AQP4 was also detected on reactive microglia following LPS injection in rats, but the significance of this expression is poorly understood (Tomás-Camardiel et al., 2004). AQP4 is also upregulated in vasogenic edema, where it occurs as a result of BBB disruption. This suggests associations between AQP4-regulated water flux and neuroinflammation (Fukuda and Badaut, 2012). Pro-inflammatory mediators including toxic cytokines IL-1 and TNF-α released by both activated microglia and by neutrophils, have been found to release matrix metalloproteinases (MMPs) in *in vitro* astrocyte cultures (Candelario-Jalil et al., 2009; Xia et al., 2010). MMPs are primary components of the neuroinflammatory response being partially responsible for BBB disruption (Rosell et al., 2006). MMP-9 degrades agrin while MMP-3 degrades dystroglycan (Wolburg-Buchholz et al., 2009), both of which play a pivotal role in OAP formation and AQP4 assembly in astroglial end-foot membranes (Noel et al., 2009; Fallier-Becker et al., 2011). The neuroinflammation produced by ischemia leads to upregulation of MMPs which cause AQP4-OAP disruption, and possibly exacerbate BBB disturbance and worsen the edema (Rosell et al., 2006). Development of vasogenic edema also amplifies BBB disruption due to increased hydrostatic pressure. AQP4 may also have a role in neuroinflammation via edema resolution (Figure 5). Up-regulation of perivascular AQP4 causes enhanced resorption of extracellular edema fluid which eases hydrostatic pressure and BBB disruption. As a result there is less neutrophil infiltration, less pro-inflammatory cytokine production and less MMP activation (Fukuda and Badaut, 2012). Stretch-activated Cl^−^ channels expressed on microglia are activated to a lesser extent because of pressure differences due to resorption of edema fluid. This leads to less activated microglia and a decrease in pro-inflammatory cytokine release (Eder et al., 1998; Fukuda and Badaut, 2012).

It can thus be postulated that AQP4 may exacerbate cytotoxic edema during the acute stage (first few hours) of ischemia, and that during later stages of edema (7 days and later), delayed but pronounced AQP4 up-regulation may play a beneficial role by promoting edema resolution and attenuating neuroinflammation by inhibition of microglial activation and astrogliosis (Tourdias et al., 2011; Fukuda and Badaut, 2012).

AQP4 is also seen to play an inhibitory role in post-ischemic inflammation (Shi et al., 2012). The inflammatory mediators cysteine leukotrienes (CysLTs), by activation of CysLT1 and CysLT2 receptors precipitate post-ischemic inflammation (Fang et al., 2006; Zhao et al., 2011). AQP4^−/−^ mice showed more pronounced up-regulation of CysLT1 and CysLT2 expression after 30 min of MCAO and enhanced inflammatory response (Shi et al., 2012). Inflammatory reaction was also exacerbated in these mice which also had a reduced number of CD4+ and CD25+ regulatory T cells (Chi et al., 2011). This may suggest that the absence of AQP4 disrupts these immunosuppressive regulators (Shi et al., 2012).

## Aquaporin 1

AQP1 is expressed on the apical surface of the choroid plexus (Gonen and Walz, 2006), which forms the border between the CSF and blood, the major fluid compartments in the brain (Masseguin et al., 2000; Papadopoulos et al., 2007). It is most notably absent in the cerebrovascular endothelial cells, except in the circumventricular organs (Nielsen et al., 1993; Wilson et al., 2010). In the spinal cord, AQP1 is expressed in the ependymal cells lining the central canal, but more robustly in the sensory fibers of the superficial laminae of the dorsal horn (Oshio et al., 2005; Shields et al., 2007; Nesic et al., 2008).

## AQP1 and cerebrospinal fluid

Due to the location of its expression, AQP1 is believed to have a role in CSF secretion under physiological conditions (Amiry-Moghaddam and Ottersen, 2003c). CSF production is driven by an osmotic gradient brought about by Cl^−^/HCO^3−^ and Na^+^/H^+^ exchangers located in basolateral membranes of the choroid plexus (Keep et al., 1994; Wu et al., 1998; Nakamura et al., 1999). AQP1 on apical membranes may explain the high osmotic-diffusional water permeability ratio in the choroid epithelium, indicating the existence of an aqueous pathway (Oshio et al., 2005). In AQP1-null mice, osmotic water permeability in the choroid plexus is reduced by five-fold compared to that in wild-type mice. This translates to a 50% reduction in intracranial pressure (Papadopoulos and Verkman, 2013) and may have important implications in ischemia. That AQP1 is not the sole player in CSF secretion is demonstrated by a mere 25% reduction in CSF secretion rate in AQP1-null mice compared to wild-type (Oshio et al., 2005). The role of this water channel in CSF production is still ambiguous and to date, studies using AQP1^−/−^ mice have not yet established it either (MacAulay and Zeuthen, 2010).

While AQP1 expression in astrocytes is linked to CSF formation, its neuronal expression may be linked to neuroplasticity and repair because of its co-localization with growth-associated protein-43 (GAP-43) (Nesic et al., 2008). AQP1 is implicated in malignant brain tumors, where it becomes expressed in the microvascular endothelium and reactive astrocytes in rodents and humans. This expression may aid in augmenting the BBB water permeability of such tumors (Papadopoulos et al., 2007). Expression of AQP1 on reactive astrocytes points to an unanticipated function of AQP1 in cell migration. This may have implications in recovery from stroke but the relationship between the two has not been investigated (Saadoun et al., 2005; Verkman, 2005). AQP1 inhibitors could be potentially beneficial in counteracting excessive CSF accumulation in ischemic stroke and in hydrocephalus or intracranial hypertension (Tait et al., 2008; Huber et al., 2012).

## Aquaporin 9

AQP9 forms part of the aquaglyceroporin family with which it shares a broad range of permeability to diverse molecules such as water, glycogen, lactate, urea, purines, pyrimidines, and monocarboxylates (Yamamoto et al., 2001). Expressed mostly in the liver and in the CNS, AQP9 is also expressed in the spinal cord and retina. In contrast to AQP4, which is expressed primarily in the foot processes of astrocytes, AQP9 is reported to be expressed throughout the astrocyte cell bodies and processes in the brain. AQP9 was also detected in cerebrovascular endothelia, astrocytes of the glia limitans, white matter tracts and tanyctes in circumventricular organs (Elkjaer et al., 2000; Badaut et al., 2001; Nicchia et al., 2001; Badaut and Regli, 2004). AQP9 is also expressed in neurons but appears to be concentrated in catecholaminergic and glucose-sensitive neurons (Badaut and Regli, 2004) and mitochondria (Amiry-Moghaddam et al., 2005), raising the possibility that AQP9 is involved in brain energy metabolism. AQP9 regulation is thought to involve PKC and PKA pathways. AQP9 mRNA is up-regulated by PKA and down-regulated by PKC pathways in astrocyte cultures. However, direct regulation by phosphorylation has not been demonstrated to date (Badaut and Regli, 2004; Papadopoulos et al., 2007; Badaut, 2010).

The role of AQP9 is still unclear although it is implicated in several pathways and its expression is altered in pathophysiological conditions (Badaut and Regli, 2004). Its permeability to glycogen and lactate suggests that it is involved in metabolite diffusion (Badaut et al., 2002; Papadopoulos et al., 2007; Badaut, 2010). AQP9 permeability varies with changes in pH, such that permeability to lactate increases four-fold when pH decreases to 5.5 (Tsukaguchi et al., 1998). Expression of AQP9 is affected by metabolic challenge. In diabetes for instance, this channel is over-expressed in neurons (Badaut, 2010; Badaut et al., 2011). Channel expression is also enriched in mitochondrial membranes, but not in all brain mitochondria, showing a preference for astrocyte mitochondria and subsets of neurons (Amiry-Moghaddam et al., 2005). AQP9 is implicated in control of extracellular milieu from its expression in astrocyte cell processes and cell bodies without vasculature contact. It is also thought to play a buffer role in active neurons and axons (Badaut et al., 2001).

## AQP9 in cerebral ischemia

AQP9 is up-regulated in reactive astrocytes at the border of the infarct after transient MCAO in mice but mainly in the cortex, ventral pallidum and nuclei of the amygdala (Badaut et al., 2001) with significant induction after 24 h and further increase over time (de Castro Ribeiro et al., 2006; Badaut, 2010). Expression was greatest at 7 days after stroke, indicating a role in reactive astrocyte and glial scar formation (de Castro Ribeiro et al., 2006). The similar regional distribution of up-regulated AQP4 indicates a potential involvement of both AQPs in water fluxes and edema formation (Marmarou, 2007; Badaut et al., 2011). However, no association with edema was found for AQP9 suggesting that in ischemia, AQP9 may have a more significant role as a metabolite channel (Bertrand et al., 1992; Schulz et al., 2000; Frykholm et al., 2001; Kuo et al., 2003). In contrast to AQP4, AQP9 is not polarized on astrocyte end-feet and its pattern of expression is thought to support a role in the ‘lactate shuttle’ model as proposed by Pellerin and Magistretti (1994). Transport of lactate into mitochondria is considered to be one of the major roles of AQP9. When ischemic lactic acidosis is exposed to the relatively high pH of the mitochondrial membrane, it is deprotonated and serves to attenuate the proton gradient across the membrane. This concentration gradient is particularly pronounced in hypoxia-ischemia, and favors lactate uptake through AQP9 in mitochondria (Amiry-Moghaddam et al., 2005). AQP9 may favor lactate clearance from the ECS and facilitate movement of lactate between astrocytes and neurons, thus promoting neuronal recovery by aiding in the provision of the energy substrate (Badaut, 2010). Up-regulation of AQP9 after ischemia could therefore be viewed as a cell survival mechanism and may represent a novel pathway in ischemic pre-conditioning (Amiry-Moghaddam et al., 2005). However, the precise roles of AQP9 are speculative (Badaut, 2010) and the lack of suitable and more specific AQP9 antibodies hinders further progress (Papadopoulos et al., 2007; Rojek et al., 2007).

## AQP3, AQP5, and AQP8

AQP3, 5, and 8 have been found in neuronal and astrocyte cultures, and AQP8 has also been found in oligodendrocytes (Yamamoto et al., 2001; Badaut et al., 2002). AQP5 expression may be attenuated during hypoxia and up-regulated after re-oxygenation. It may thus partake in resolution or progression of edema (Yamamoto et al., 2001). The tandem up-regulation of both AQP5 and APQ4 may be a mechanism by which cerebral edema is attenuated by mild hypothermia under hypoxic conditions (Fujita et al., 2003). The physiological roles of these AQP members and their implications in stroke pathophysiology remain to be elucidated (Badaut et al., 2002).

## Practical implications and future direction

Ischemic stroke is a major cause of long-term disability in humans, but effective treatments are lacking. Studies on AQPs provide a compelling rationale for development of AQP modulators that have the potential for improving ischemic pathologies (Table 1). Although some potential AQP modulators have been identified, challenges and speculation still abounds (Papadopoulos and Verkman, 2013). Development of potential therapeutic agents targeted at AQPs for management of ischemia is only successful when the type of stroke and the timing of intervention are taken into consideration. Furthermore, the variety of AQP expression patterns adds to the complexity of treating this disease (Badaut et al., 2011).

**Table 1:** **Summary of different treatments with potential for clinical use in ischemia-evoked cerebral edema and their effects on AQP expression**.

**Treatment**	**AQP expression after ischemia and treatment**	**Ischemic model**	**References**
Acetazolamide	Decreases AQP4 and AQP1 permeability	Pure proteins reconstituted into liposomes; Xenopus oocytes	Ma et al., 2004; Tanimura et al., 2009
Anti-miR-320a	Increases AQP4 and AQP1 expression	Rat transient MCAO, 60 min	Sepramaniam et al., 2010
AqB013	Non-selective AQP1/APQ4 inhibitor	Xenopus oocytes	Migliati et al., 2009; Yool et al., 2009
Arginine vasopressin V1 (AVPV1) antagonist	Increases AQP4 expression	Mouse transient MCAO, 60 min	Liu et al., 2010
Bumetanide	Decreases AQP4 permeability	Mouse transient MCAO, 60 min	Migliati et al., 2009
Edaravone	Decreases AQP4 expression	Rat transient MCAO, 90 min	Kikuchi et al., 2009
EZA (6-ethoxy-benzothiazole-2-sulfonamide)	Decreases AQP4 permeability	Xenopus oocytes	Huber et al., 2007
Furosemide	Decreases AQP4 and AQP1 permeability	Xenopus oocytes	Migliati et al., 2009; Ozu et al., 2011
miR-320a	Decreases AQP4 and AQP1 expression	Rat transient MCAO, 60 min	Sepramaniam et al., 2010
Neuregulin	Decreases AQP4 expression	Rat transient MCAO, 90 min	Li et al., 2008
NSC164914 (tributyl-(2,4,5-trichlorophenoxy)stannane); NSC168597 (tributyl-chloroplumbane)	Decrease AQP4 and AQP1 permeability	Fluorescence-based assays of volume changes in primary cultures of mouse astrocytes	Mola et al., 2009
PMA (phorbol 12-myristate 13-acetate)	Decreases AQP4 expression	Rat transient MCAO, 120 min	Kleindienst et al., 2006; Fazzina et al., 2010
Picroside II	Decreases AQP4 expression	Rat BCCAO 60, 90, 120, 150 min	Li et al., 2010, 2013
Piroxicam	Decreases AQP4 expression	Rat transient MCAO, 60 min	Bhattacharya et al., 2012
Probenecid	Decreases AQP4 expression	Mouse transient MCAO, 60 min	Xiong et al., 2014
Propofol	Decreases AQP4 expression	Rat transient MCAO, 90 min	Zheng et al., 2008
TEA (Tetraethylammonium)	Decreases AQP1 permeability	Xenopus oocytes	Brooks et al., 2000
TGN-020-(2-(nicotinamoyl)-1,3,4-thiadazole)	Decreases AQP4 permeability	Mouse transient MCAO, 120 min	Igarishi et al., 2011

*Abbreviations: MCAO, Middle Cerebral Artery Occlusion; BCCAO, Bilateral Common Carotid Artery Occlusion*.

A major outcome measure in the treatment of ischemia is the abatement of the edematous process (Badaut et al., 2011). AQP modulators are expected to decrease brain water content and thereby decrease intracranial pressure to improve cerebral perfusion, alleviate herniation and decrease the risk of death (Papadopoulos and Verkman, 2008). They would need to be administered for a few days when edema persists and would also be expected to act acutely. Candidate drugs need to possess good pharmacology, be stable and non-toxic and capable to circumvent the BBB. Most of the information on potentially viable treatment options concerns the channel AQP4. Because of its dual action, inhibitors or activators of AQP4 could theoretically be applied at different stages of stroke to exert specific actions. For instance, AQP4 inhibitors may be beneficial in the early course of stroke (cytotoxic phase) but potentially detrimental later on (vasogenic phase) (Papadopoulos and Verkman, 2008).

There are no specific AQP4 inhibitors with clinical usefulness, although AQP4 down-regulation can be achieved by inhibition of RNA or modulators based on the crystal structure of AQP (Papadopoulos and Verkman, 2007; Saadoun and Papadopoulos, 2010). Drug discovery is also possible through rigorous morphological assays, water-based transport screens and nanotechnology-based screens (Frigeri et al., 2007). siRNA strategy permitted to show the potential of a specific AQP4 inhibitor in a rat model of juvenile traumatic brain injury. This study showed a decrease in edema formation and functional recovery post injury (Fukuda et al., 2013b) in parallel to neuroimaging approach findings (Manley et al., 2000). One area of recent progress is the immunosuppressive approach to block the onset of neuromyelitis optica by a combination of methods based on anti-AQP4 antibody binding to pathogenic autoantibodies and screening of blockers of the autoantibody-AQP4 interaction (Verkman et al., 2013).

Compounds that have been tested include bumetanide (NKCC1 inhibitor), a clinically safe diuretic, which is thought to block the AQP4 channel and water permeability in *X. laevis* oocytes (Migliati et al., 2009). In experimental rodent models of ischemia this prevents edema formation through decreases in AQP4 expression. However, this effect may also be attributed to the potential blockade of the Na-Cl-K co-transporter in endothelial cells (Migliati et al., 2010; Badaut et al., 2011). Acetazolamide and sulphonamide, as well as several other anti-epileptic drugs have reported to inhibit AQP4 expressed in *X. laevis* oocytes (Huber et al., 2007, 2009). In another study, AQP4-mediated water conduction in proteoliposomes was reversibly inhibited by acetazolamide (Tanimura et al., 2009). In a recent rat focal ischemia model, pre-treatment with piroxicam was shown to be a potent AQP4 regulator, rendering neuroprotection (Bhattacharya et al., 2013).

Also reported to confer neuroprotective effects in ischemia is erythropoietin. Intracerebroventricular administration 24 h prior to induction of ischemia resulted in a 47% reduction of infarction in mice subjected to MCAO (Bernaudin et al., 1999). Its modes of action include a transient increase in intracellular Ca^2+^ (Morishita et al., 1997) and attenuation of NO-induced cell death (Sakanaka et al., 1998). However, further studies are needed to further demonstrate the connection of these in relation to ischemia amelioration.

A potentially viable compound which is postulated to interact with AQP4 and which may be useful in ischemic stroke is edaravone, a free radical scavenger that readily crosses the BBB. In a rat transient focal ischemia model, it significantly reduced the area of infarction and improved neurological scores when administered after 24 h of reperfusion. Furthermore, edaravone markedly reduced AQP4 immunoreactivity and protein levels in the cerebral infarct area. It is postulated that by suppression of AQP4 expression, edaravone exerts its influence to reduce cerebral ischemic injury (Kikuchi et al., 2009). Similarly antioxidant administration of melatonin showed reduced AQP4 expression in a study involving hypoxic rats (Kaur et al., 2006). Propofol has also been reported to down-regulate AQP4 expression. In a model of MCAO and reperfusion, infusion of propofol prior to ischemia decreased brain edema and reduced over-expression of AQP4. This may be mediated by altered regulation of the PKC pathway in the brain, or attenuation of extracellular glutamate levels (Zheng et al., 2008). In a focal ischemia mouse model with reperfusion, administration of Neuregulin-1β (NRG-1β) confered neuroprotection through regulation of the activation of AQP4 and inhibition of the mitochondrial apoptosis pathway. The mechanism by which this occurs however, remains partly elusive (Li et al., 2008).

Since AQP4 aids in migration of reactive astrocytes toward an injury site, candidate AQP4 blockers may augment neuronal generation by attenuating reactive gliosis and hence mitigate potentially detrimental glial scar formation (Saadoun and Papadopoulos, 2010). Migration can also be modulated by altering extracellular osmolarity or transcellular osmotic gradients, since AQP4 supports water flux into and out of migrating astrocytes (Papadopoulos and Verkman, 2013).

Agmatine attenuated AQP1 expression in a mouse model of transient ischemia. This translates into decrease of cerebral edema and brain swelling, possibly through reduction of matrix metalloproteinases-2 and -9 (Kim et al., 2008), which are associated with BBB disruption and could thus offer another possible therapeutic approach in treatment of brain edema (Kim et al., 2010).

## Conclusion

Substantial advancements have been made in the field of AQP studies and their role in several pathophysiologies. Regulation of water transport constitutes an integral part of ischemic stroke amelioration and thus continuous efforts are required in the hope of elucidating further mechanisms that could improve outcomes of this disease. Careful application of various AQP modulators at different phases of stroke has the potential of amplifying favorable outcomes and of attenuating undesirable ones, making them clinically highly relevant. Finally, the development of new tools to identify selective modulators with subtype specificity and affinity for select AQPs is poised for exciting progress in basic life sciences and medicine.

## Author contributions

JV and MV conceived the work, drafted, designed and revised critically. CZ, GG, and RM acquired and compiled information. All authors wrote and revised the manuscript.

### Conflict of interest statement

The authors declare that the research was conducted in the absence of any commercial or financial relationships that could be construed as a potential conflict of interest.
